# Cortical Gyrification Morphology in Adult Males with Mild Traumatic Brain Injury

**DOI:** 10.1089/neur.2021.0032

**Published:** 2022-08-09

**Authors:** Avideh Gharehgazlou, Rakesh Jetly, Shawn G. Rhind, Amy C. Reichelt, Leodante Da Costa, Benjamin T. Dunkley

**Affiliations:** ^1^ Neurosciences and Mental Health, The Hospital for Sick Children (SickKids) Research Institute, Toronto, Ontario, Canada.; ^2^ Bloorview Research Institute, Holland Bloorview Kids Rehabilitation Hospital, Toronto, Ontario, Canada.; ^3^ Directorate of Mental Health, Canadian Forces Health Services HQ, Ottawa, Ontario, Canada.; ^4^ Defence Research and Development Canada–Toronto Research Centre, Toronto, Ontario, Canada.; ^5^ Faculty of Kinesiology and Physical Education, University of Toronto, Toronto, Ontario, Canada.; ^6^ Sunnybrook Health Sciences Centre, Toronto, Ontario, Canada.; ^7^ Institute of Medical Science, Faculty of Medicine, University of Toronto, Toronto, Ontario, Canada.; ^8^ Department of Surgery, University of Toronto, Toronto, Ontario, Canada.; ^9^ Department of Diagnostic Imaging, The Hospital for Sick Children (SickKids) Research Institute, Toronto, Ontario, Canada.; ^10^ Department of Medical Imaging, University of Toronto, Toronto, Ontario, Canada.; ^11^ Faculty of Health and Medical Sciences, University of Adelaide, Adelaide, South Australia, Australia.

**Keywords:** cortical gyrification, *l*GI, mean curvature, mild traumatic brain injury, mTBI, structural MRI

## Abstract

Cortical gyrification, as a specific measure derived from magnetic resonance imaging, remains understudied in mild traumatic brain injury (mTBI). Local gyrification index (*l*GI) and mean curvature are related measures indexing the patterned folding of the cortex,ml which reflect distinct properties of cortical morphology and geometry. Using both metrics, we examined cortical gyrification morphology in 59 adult males with mTBI (*n* = 29) versus those without (*n* = 30) mTBI in the subacute phase of injury (between 2 weeks and 3 months). The effect of IQ on *l*GI and brain-symptom relations were also examined. General linear models revealed greater *l*GI in mTBI versus controls in the frontal lobes bilaterally, but reduced *l*GI in mTBI of the left temporal lobe. An age-related decrease in *l*GI was found in numerous areas, with no significant group-by-age interaction effects observed. Including other factors (i.e., mTBI severity, symptoms, and IQ) in the *l*GI model yielded similar results with few exceptions. Mean curvature analyses depicted a significant group-by-age interaction with the absence of significant main effects of group or age. Our results suggest that cortical gyrification morphology is adversely affected by mTBI in both frontal and temporal lobes, which are thought of as highly susceptible regions to mTBI. These findings contribute to understanding the effects of mTBI on neuromorphological properties, such as alterations in cortical gyrification, which reflect underlying microstructural changes (i.e., apoptosis, neuronal number, or white matter alterations). Future studies are needed to infer causal relationships between micro- and macrostructural changes after an mTBI and investigate potential sex differences.

## Introduction

Mild (mTBI) traumatic brain injury (TBI), the most common form of TBI,^1^ is a serious public health issue.^2^ Defined as transient impairment to mental functioning, which may or may not include loss of consciousness, it is caused by biomechanical forces acting on the brain.^3^ Despite spontaneous recovery in the majority of cases,^3^ a significant minority experience persistent functional problems.^4,5^ Although neuroimaging has been used post-mTBI to understand the neurobiology of injury, it is not currently diagnostic or prognostic for mTBI. Rather, other protocols, such as the Glasgow Coma Scale (GCS),^6^ clinical history, and information regarding the injurious event, are used for diagnostic purposes.^7^

Magnetic resonance imaging (MRI) studies have extensively contributed to our understanding of mTBI through reports of structural^8–10^ and functional^11^ dysregulation. The assessment of cortical gyrification—a calculation of cortical architecture derived from MRI—has been limited in mTBI.^12,13^ Cortical gyrification refers to the convex and concave patterning of the cerebral cortex, which begins pre-natally and intensifies during the third trimester,^14^ at which point the cortex is transformed from a lissencephalic into a gyrencephalic structure. Mirroring other metrics of cortical gray matter (volume, thickness, and surface area), cortical gyrification exhibits an inverted-U developmental trajectory, peaking at ∼2–3 years of age,^15,16^ and then declining sharply during childhood and adolescence^17^ and gradually decreasing through adulthood.^18,19^ Moreover, recent imaging studies suggest that gyrification is a potential marker for age-related brain and cognitive decline beyond midlife.^20^

Environmental factors may contribute to cortical gyrification morphology (i.e., meditation^21^ and physical training^22^); however, mTBI can be framed as an insidious environmental factor that may also impact gyrification and plasticity—given that neurotrauma is associated with inflammation/edema,^23^ apoptosis,^24^ and demyelination—presenting as alterations to gray^10,25^ and white matter.^26^ To date, two studies^12,13^ have examined cortical gyrification in mTBI using separate morphological metrics of gyrification: local gyrification index (*l*GI) and mean curvature. *l*GI quantifies the degree of local gyrification by estimating the amount of the unexposed surface hidden within sulci and has been used in studies of neuropsychiatric^27,28^ and neurodevelopmental disorders.^29^ Mean or extrinsic curvature (computed as the average of the two principal curvatures: maximal, K_1_ and minimal, K_2_ curvatures) captures the extrinsic qualities of a surface^30^ (i.e., degree of sharpness^31^) and is a related, but distinct, property of cortical geometry when compared with *l*GI.^32^ Rather, *l*GI is akin to intrinsic or Gaussian curvature (product of the two principal curvatures), with the difference being that it captures a wider area (50-mm diameter).^32^ Although mean curvature and *l*GI are related measures of the gyrification of the cortex, it is important to consider their methodological differences when determining cortical morphology and geometry.

In this study, we investigated cortical gyrification morphology in a cohort of male adults with and without mTBI using both the *l*GI and mean curvature measures to assess associations between *l*GI and mTBI severity during the acute/subacute phase and functional symptomology. Previous studies using this cohort have determined changes to structural morphology in acute/subacute mTBI, using various indices including decreased cortical volume and cortical thickness.^10^ Given the accepted association between cortical thickness and neuronal number,^33,34^ and mean curvature as a reliable measure of underlying white matter atrophy,^35^ we predict that mTBI will result in a reduction in gyrification and curvature and provide a better understanding of morphological dysregulation in the cortex after mTBI. To test this prediction, we examined 1) *l*GI group differences with and without controlling for the effects of mTBI severity, symptoms, and IQ and 2) mean curvature group differences.

## Methods

### Participants

Adult males (*N* = 59; 29 mTBI, 30 control) 20–45 years of age were recruited for this study. Participants with mTBI were recruited from a level 1 trauma center (Sunnybrook Health Sciences Centre, Toronto, Ontario, Canada) in Toronto, with a diagnosis made by a physician. Inclusion criteria were: an mTBI within the previous 3 months (mean time post-injury at scan = 33.33 days), no reported history of mTBI, and a normal head computed tomography scan at admission. Symptomatology was not a prerequisite for inclusion, but when present was limited to loss of consciousness of no more than 30 min, post-traumatic amnesia, alterations of consciousness, and/or confusion of no more than 24 h and GCS >13 in the first 24 h after injury.^6^

Control group participants were recruited from the local community and through flyers posted at the hospital. Exclusion criteria included: self-reported history of neurological, psychological, and psychiatric disorders and previous concussive TBI that resulted in a transient alteration of mental function. Exclusion criteria for both groups included taking anticonvulsant medications, benzodiazepines, and gamma-aminobutyric acid antagonists, contraindication to MRI, or gross neurostructural abnormalities and/or significant artefacts in their MRI scan.

This study was approved by and conducted in accordance with the research ethics boards of The Hospital for Sick Children (Toronto, Ontario, Canada) and Sunnybrook Health Sciences Centre. Informed written consent was obtained from all participants.

### Cognitive-behavioral assessment

Intelligence levels were assessed using Wechsler Abbreviated Scale of Intelligence (WASI).^36^ mTBI symptomology and severity were assessed using the Sports Concussion Assessment Tool 2 (SCAT2).^37^ These assessments were administered to all participants on the day of MRI scanning. For our brain-behavior analyses, we obtained the “total symptom” and “symptom severity” scores from SCAT2. The symptom score refers to the total number of symptoms a participant has, and a maximum possible score would be 22, whereas the severity score refers to the severity of the symptoms a participant has—obtained through the summation of all scores—yielding a maximum score of 132.

### Neuroimaging data acquisition parameters

T_1_-weighted images were obtained using a 3 Tesla Siemens Trio MRI scanner (with a 12-channel head coil, MAGNETOM Tim Trio; Siemens AG, Erlangen, Germany) at the Hospital for Sick Children. The sequence was a three-dimensional (3D) sagittal magnetization-prepared rapid gradient echo with repetition time = 2300 ms, echo time = 2.96 ms, inversion time = 900 ms, and flip angle = 90 degrees. A field of view of 240 × 256 mm with 192 slices yielded 1-mm isotropic voxels. Motion restriction and stabilization of the head during imaging were attained with foam padding.

### Image processing

FreeSurfer software version 6.0^38^ was used for image processing and reconstruction, as described in a previous work.^39^ Briefly, T_1_-weighted images were registered to MNI305 atlas before intensity normalization, skull stripping, and white matter segmentation steps. A cortical surface mesh was computed for each individual scan. Pial (gray matter/cerebral spinal fluid boundary) and white (gray-white matter boundary) surfaces were then differentiated. Using spherical registration, individual scans were registered to FreeSurfer's template space, called fsaverage. Last, cortical segmentation based on the Desikan-Killany atlas was performed.

### Quality control

Quality control on FreeSurfer output was performed based on ENIGMA Cortical Quality Control Protocol 2.9 (April 2017; http://enigma.ini.usc.edu) through visual inspection of gray-white matter segmentation and cortical labeling. Manual troubleshooting was performed when needed. Three participants (2 mTBI, 1 control) were excluded, resulting in 27 mTBI and 29 controls in the final sample (Table 1).

**Table 1.** Participant Demographics

	Control group (*n* = 29)	mTBI group (*n* = 27)	p value
Mean age (years) ± SD [range]	28.0 (4.63) [20.55–39.03]	29.99 (6.43) [20–44]	N.S.
Mean SA ± SD [range]	221,861.52 (17,242.49) [193,840–254,651]	225,430.11 (18,928.03) [198,343–291,751]	N.S.
Mean eTIV ± SD [range]	1,624,688.53 (119,128.61) [1,369,627.11–1,823,065.34]	1,664,684.56 (130,850.23) [1,439,530.16–1,971,652.11]	N.S.

SD, standard deviation; SA, surface area (mm^2^); eTIV, estimated total intracranial volume (mm^3^); mTBI, mild traumatic brain injury; N.S., no significant differences between groups as indicated by *t*-tests.

### Local gyrification index and mean curvature

*l*GI was computed on FreeSurfer by quantifying the degree of gyrification locally at thousands of vertices across each hemisphere.^32^ *l*GI is a 3D measure of the ratio between the area of an estimated circular region of interest (ROI; 25-mm radius) on the pial surface and the area of the corresponding ROI on the outer surface (i.e., *l*GI of 5, highly folded cortex; *l*GI of 1, a smooth cortex).

Mean curvature was computed by default on FreeSurfer after the execution of the “recon-all” command. At each vertex on the surface, mean curvature was calculated as the average of the two principal curvatures, K_1_ and K_2_, maximum and minimum curvatures, respectively.

### Statistical analyses

To compare *l*GI and mean curvature values between groups, we undertook a whole-brain approach and conducted general linear models (GLMs) on the Query, Design, Estimate, Contrast (QDEC) application on FreeSurfer, utilizing the DODS (different offset, different slope) design matrix. For *l*GI analyses, we controlled for the effect of surface area given the strong positive correlation between the *l*GI measure and surface area,^40^ and we did not apply any smoothing.^41^ For mean curvature analyses, we controlled for the effect of estimated total intracranial volume (eTIV), similar to previous curvature studies,^31,42,43^ and applied a 20–full width at half maximum smoothing kernel. The effect of age was controlled for in both *l*GI and mean curvature analyses, and all covariates were demeaned. Last, Monte-Carlo z-simulations (*p* < 0.05) correction was used.

To examine the relation of IQ with *l*GI, we repeated our *l*GI main analyses (with the identical procedures described above) on the subset of participants with available IQ scores (23 mTBI, 22 controls). Briefly, including this covariate resulted in minimal changes to the between-groups contrast without the covariate, showing that IQ played a minimal role in the results. To explore the association between mTBI severity and symptoms with *l*GI, we undertook a whole-brain approach and conducted GLMs on QDEC (DODS) while controlling for the effects of diagnosis, age, and surface area (latter two demeaned). No smoothing was applied, and Monte-Carlo (*p* < 0.05) correction was used.

## Results

### Participant demographics

Independent *t*-tests conducted on the whole sample (*n* = 56) indicated no significant group differences in age, surface area, or eTIV (Table 1).

### Mild traumatic brain injury is associated with increased frontal and reduced temporal local gyrification index

**FIG. 1.**
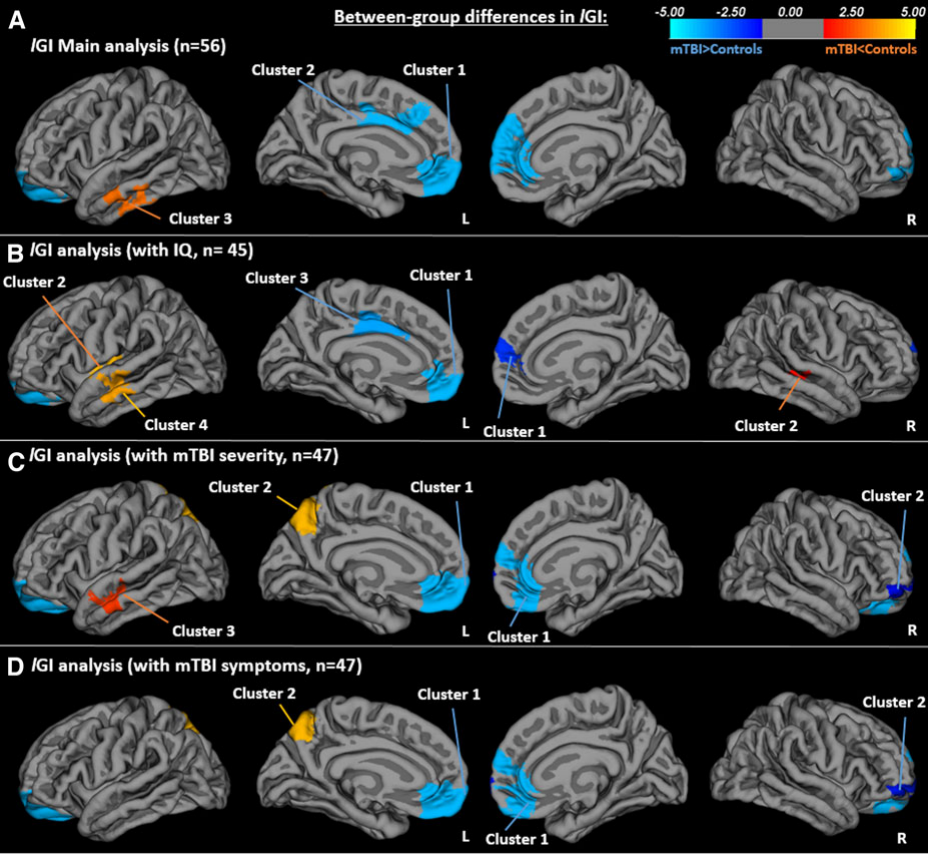
(**A**) Local gyrification index (*l*GI) main analysis, *n* = 56 (*L*: cluster 1 peak: lateral orbitofrontal, *p* = 0.0001; cluster 2 peak: superior frontal, *p* = 0.0001; cluster 3 peak: middle temporal, *p* = 0.001; *R*: peak: parsorbitalis, *p* = 0.0001). (**B**) *l*GI analysis with IQ in the model, *n* = 45 (*L*: cluster 1 peak: lateral orbitofrontal, *p* = 0.0001; cluster 2 peak: superior temporal, *p* = 0.0001; cluster 3 peak: superior frontal, *p* = 0.0002; cluster 4 peak: middle temporal, *p* = 0.0002; *R*: cluster 1 peak: rostral anterior cingulate, *p* = 0.0089; cluster 2 peak: middle temporal, *p* = 0.03420). (**C**) *l*GI analysis with mTBI severity in the model, *n* = 47 (*L*: cluster 1 peak: lateral orbitofrontal, *p* = 0.0001; cluster 2 peak: precuneus, *p* = 0.0001; cluster 3 peak: middle temporal, *p* = 0.0071; *R*: cluster 1 peak: rostral anterior cingulate, *p* = 0.0001; cluster 2 peak: pars orbitalis, *p* = 0.03490). (**D**) *l*GI analysis with mTBI symptoms in the model, *n* = 47 (*L*: cluster 1 peak: medial orbitofrontal; cluster 2 peak: precuneus, both *p*s = 0.0001; *R*: cluster 1 peak: rostral anterior cingulate, *p* = 0.0001; cluster 2 peak: rostral middle frontal, *p* = 0.0225).

GLMs yielded significant group differences with greater bilateral *l*GI in mTBI compared to controls in the frontal lobes (Fig. 1A; left: cluster 1 peak: lateral orbitofrontal, cluster 2 peak: superior frontal; right: cluster peak: pars orbitalis; all *p*s = 0.0001), but reduced *l*GI in mTBI in a cluster located in the left temporal lobe (Fig. 1A; cluster 3 peak: middle temporal; *p* = 0.0012).

We examined *l*GI in participants with available IQ scores (WASI; *n* = 23 mTBI, *n* = 22 controls; Fig. 1B). In the left hemisphere, we found greater *l*GI in mTBI compared to controls in the lateral and medial orbitofrontal cortex (cluster 1 peak: lateral orbitofrontal; *p* = 0.0001) and posterior cingulate gyrus (cluster 3 peak: superior frontal; *p* = 0.0002; note: this cluster does not extend to the medial superior frontal lobe as it did in the main analysis). Reduced *l*GI in mTBI was observed in the temporal lobe, but unlike the main analysis, this effect was then shifted superiorly to include the middle (cluster 4 peak: middle temporal; *p* = 0.0002) and superior temporal gyri extending to the insular region (cluster 2 peak: superior temporal; *p* = 0.0001; Fig. 1B), rather than the inferior temporal gyri (Fig. 1A). We found greater *l*GI in mTBI in the right medial and lateral prefrontal cortices (cluster 1 peak: rostral anterior cingulate; *p* = 0.0089); however, these clusters were much smaller than those identified in the main analysis. A new right hemisphere cluster was identified in the lateral temporal lobe (cluster 2 peak: middle temporal; *p* = 0.03420) depicting reduced *l*GI in mTBI. Significant positive associations between *l*GI and IQ and interaction effects are reported in the Supplementary Materials (Supplementary Fig. S2).

### Mild traumatic brain injury severity and symptoms are associated with increased frontal and reduced temporal and parietal local gyrification index

To determine associations of *l*GI with mTBI severity (SCAT2; *N* = 47; mTBI, *n* = 27; control, *n* = 20), we further examined group differences in *l*GI with severity included in the model. We found greater *l*GI in mTBI in a left orbitofrontal cluster (Fig. 1C; cluster 1 peak: lateral orbitofrontal; *p* = 0.0001) and reduced *l*GI in mTBI in a left temporal lobe cluster (cluster 3 peak: middle temporal; *p* = 0.0071). The significant medial frontal lobe cluster no longer reached significance; however, a significant cluster was found in the superior parietal lobe (cluster 2 peak: precuneus; *p* = 0.0001) depicting reduced *l*GI in mTBI. Right hemisphere analysis showed greater *l*GI in mTBI in clusters located in the frontal lobe (cluster 1 peak: rostral anterior cingulate, *p* = 0.0001; cluster 2 peak: pars orbitalis, *p* = 0.03490). Significant associations between *l*GI and mTBI severity are reported in the Supplementary Materials (Supplementary Fig. S1A).

We also examined group differences in *l*GI (*n* = 47) with symptom number included in the model. Greater *l*GI in mTBI was observed in a left orbitofrontal cluster (Fig. 1D; cluster 1 peak: medial orbitofrontal; *p* = 0.0001). However, the significant temporal lobe and medial superior frontal lobe clusters in the main analysis did not reach significance. Instead, reduced *l*GI in mTBI was observed in a superior parietal cluster (cluster 2 peak: precuneus; *p* = 0.0001). In the right hemisphere, greater *l*GI in mTBI was found in clusters located in the frontal lobe (cluster 1 peak: rostral anterior cingulate, *p* = 0.0001; cluster 2 peak: rostral middle frontal, *p* = 0.0225). Significant associations between *l*GI and mTBI symptoms are reported in the Supplementary Materials (Supplementary Fig. S1B).

**FIG. 2.**
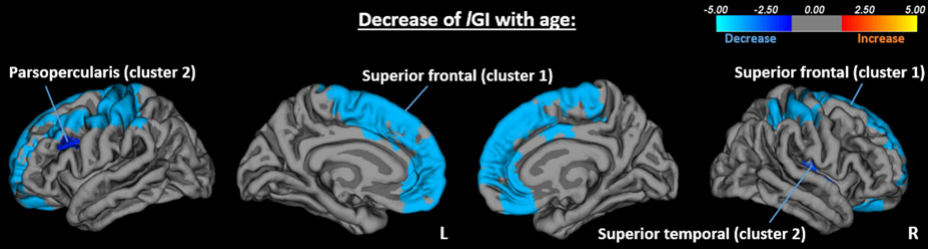
Decrease of *l*GI with age in left (cluster 1 peak: superior frontal, *p* = 0.0001; cluster 2 peak: pars opercularis, *p* = 0.013) and right (cluster 1 peak: superior frontal, *p* = 0.0001; cluster 2 peak: superior temporal, *p* = 0.0033) hemispheres.

A significant decrease of *l*GI with age was found across groups in two clusters covering the left frontal and parietal lobes (Fig. 2; cluster 1 peak: superior frontal, *p* = 0.0001; cluster 2 peak: pars opercularis, *p* = 0.013) and two clusters covering all lobes, except the occipital on the right hemisphere (cluster 1 peak: superior frontal, *p* = 0.0001; cluster 2 peak: superior temporal, *p* = 0.0033; Fig. 2). No significant group-by-age interaction effects were observed.

### Mean curvature

**FIG. 3.**
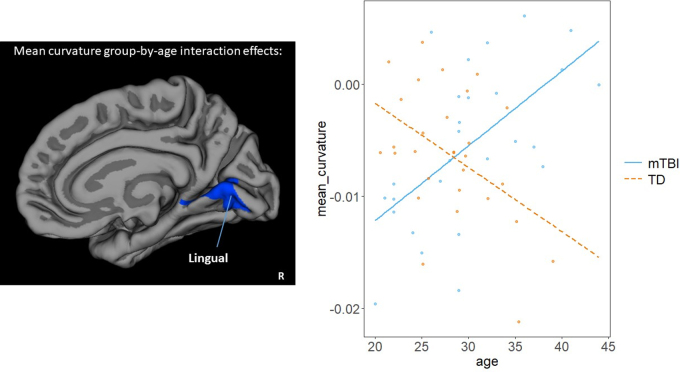
Mean curvature group-by-age interaction effects (peak: lingual; *p* = 0.006).

No significant main effects of group or age were found in either hemisphere (Fig. 3). However, a significant group-by-age interaction effect was observed, showing an increase of mean curvature with age in the mTBI group, but a decrease of mean curvature with age in the control group, in a cluster covering parts of the right medial occipital and temporal lobes (peak: lingual; *p* = 0.0059).

## Discussion

In this study, we used two distinct metrics of cortical gyrification to understand alterations in neuromorphology after mTBI in adult males. A striking observation was that of atypical *l*GI in mTBI in the frontal and temporal lobes—two highly susceptible regions in mTBI^44,45^—which demonstrates both dysregulated gyrification and microstructural disparities in these cohorts. The mechanisms of cortical gyrification in early brain development is yet to be elucidated, with theories highlighting the role of mechanical forces or biological factors that dictate the emergence of the characteristic gyri and sulci of the cerebral cortex. At a cellular level, increased gyrification is found in regions that have undergone higher rates of neurogenesis (and thus contain abundant cortical progenitors) compared to regions that have undergone lower rates of neurogenesis.^46^ As such, altered gyrification in persons with mTBI likely reflects alterations to neuronal populations, supported by histological examinations in pre-clinical mTBI models.^24^

Alternatively, the tension-based hypothesis of cortical gyrification proposes that mechanical tension along axons in early development results in strongly interconnected regions being pulled toward one another whereas less-interconnected regions separate, resulting in the emergence of gyri and sulci.^47^ This is reflected by associations between gyrification and underlying structural connectivity.^48^ Thus, cortical gyrification atypicalities in mTBI could reflect white matter structural connectivity disruptions, which we know is present in mTBI.^26^ Further, mTBI-evoked neuroinflammation can cause cerebral edema and/or damage to axonal cytoskeleton architecture^23^ disrupting white matter microstructure, resulting in altered structural connectivity. Thus, the atypical macrostructural alterations in gyrification observed in persons with mTBI may reflect underlying microstructural and/or cellular consequences of brain injury. However, it is important to note that there is still a lack of consensus regarding the theory that best accounts for cortical gyrification.^49,50^

Key observations of our study were region-specific increases and decreases in *l*GI in mTBI. These findings reflect aspects of Wilde and colleagues' (2021) mTBI study^13^; however, study population differences between our studies may account for differences in our observations. Wilde and colleagues' study sample included male and female adolescents (mean age = 14 years), whereas our cohort included adult males (mean age = 29 years). These contrasting findings indicate that age at injury influences both developmental effects^51^ and recovery trajectories,^52^ as well as structural morphological outcomes. For instance, it may be that pediatric mTBI affects gyrification differently compared to mTBI occurring during adulthood, reflecting dynamic changes in gyrification throughout the life span.^18,19^

In support of previous observations (Wilde and colleagues), we found that greater *l*GI in the left temporal lobe was associated with reduced mTBI severity (and symptoms). This is in support of our findings of reduced *l*GI in the lateral temporal lobe in mTBI relative to controls. Further positive associations between *l*GI-mTBI severity (and symptoms) were found in the left parietal lobe. Interestingly, with the inclusion of symptom or severity, we observed an additional left superior parietal cluster of reduced *l*GI in mTBI that overlapped with the cluster showing positive associations between *l*GI-mTBI severity and symptoms. As such, mTBI is generally associated with atypical gyrification in the left superior parietal lobe. Inclusion of symptom scores resulted in gyrification atypicalities merely in the left frontal and parietal lobes and left temporal cluster, showing that reduced *l*GI in mTBI no longer reached significance.

Inclusion of IQ yielded similar results to the main analysis with the addition of a right lateral temporal lobe cluster depicting reduced *l*GI in mTBI compared to controls. Additionally, the left temporal lobe cluster depicting reduced *l*GI in mTBI in the main analyses shifted superiorly to encompass the insular region, supporting other observations of atypicalities in the insular region post-mTBI such as gray matter volume.^53^ In agreement with findings of a positive association between gyrification and intelligence level in normative literature,^54,55^ we also found a positive association between IQ and *l*GI across groups.

Cortical gyrification undergoes dynamic changes throughout the life span, and in agreement with the widely reported findings of age-related decrease of *l*GI after toddlerhood,^18,19^ we also found a decrease of *l*GI with age across groups. The absence of significant group-by-age interaction effects may suggest a similar trajectory across groups in the age range of our cohort.

In contrast to the previous observation of increased mean curvature in mTBI compared to controls,^12^ we found no group differences or a main effect of age, with the exception of an increase of mean curvature with age in the mTBI group, but a decrease in the control group, in a right hemisphere cluster (Fig. 3). The null findings in our analyses may be explained by biological and methodological factors. For instance, no difference in mean curvature may exist between adult males with and without mTBI or that dysregulation is present but weak, or that a high degree of individual variability or small sample size of our cohort results in insufficient statistical power to detect small effects. King and colleagues (2016) included a larger sample (*n* = 85) compared to our study (*n* = 56), which may have captured more subtle characteristics of cortical curvature^31^ when compared to *l*GI.

Our study has several limitations, and the results should be interpreted with caveats. These include a small sample size, with a relatively weakly powered design to detect small effect sizes. It is also impossible to say whether these alterations result from the primary injury, are attributable to secondary or tertiary neurochemical cascades, whether they reflect a true pathological biomarker of mTBI, or are related to some other comorbid state, or even if they are purely epiphenomenological. It is also impossible to deduce whether these gyrification abnormalities existed in the pre-injurious state. With these data here, these issues are essentially unanswerable, and we hope to address these in future studies; for example, with a prospective study that uses pre-season imaging in contact sports to image changes directly after mTBI and compare against clinical groups with similar symptoms (e.g., post-traumatic stress disorder), but without TBI.

## Conclusion

In sum, our study presents a comprehensive understanding of cortical gyrification in adult males with mTBI through two complementary metrics of cortical gyrification. Our work is the first to identify cortical gyrification atypicalities in adults with mTBI in support of previous findings in pediatric cases. Future large-scale longitudinal studies, including female participants, are needed to investigate the nexus between cortical gyrification and microstructural properties to infer causal relationships between micro- and macrostructural changes after a concussion and investigate sex effects. Future longitudinal studies, with multiple time points, would shed light on patterns of recovery, which are not directly associated with symptomology. Such conclusions cannot be made with our current cross-sectional study design and need to be empirically investigated by future longitudinal efforts.

## Supplementary Material

Supplemental data

Supplemental data

## Abbreviations Used

3D: three-dimensional
eTIV: estimated total intracranial volume
GCS: Glasgow Coma Scale
GLMs: general linear models
*l*GI: local gyrification index
MRI: magnetic resonance imaging
mTBI: mild traumatic brain injury
ROI: region of interest
SCAT2: Sports Concussion Assessment Tool 2
TBI: traumatic brain injury
WASI: Wechsler Abbreviated Scale of Intelligence
