# Supplementary material for: Cortical Gyrification Morphology in Adult Males with Mild Traumatic Brain Injury
Source: Neurotrauma Rep. 2022 Aug 9;3(1):299–307. doi: 10.1089/neur.2021.0032 (PMC9438439; doi:10.1089/neur.2021.0032)
Supplement: Supplemental data [file Supp_FigS2.docx]

**Effect of IQ on *l*GI**

Across groups (n=45), a positive association between *l*GI and IQ was found in two left hemisphere medial clusters in the parietal (cluster 1 peak: precuneus, *p*=0.0001) and occipital (cluster 2 peak: pericalcarine, *p*=0.0013, Figure S2, A) lobes. Significant group-by-IQ interaction effects were also observed in clusters covering all lobes except the temporal on the left and all lobes except the frontal on the right hemisphere (Figure S2, B). Specifically, all group-by-IQ interactions, except the left frontal cluster, depicted a positive association between *l*GI and IQ in the control group but a negative association in the mTBI group. In contrast, the left frontal cluster (cluster 2) showed the opposite direction of effect (‘-‘ association between *l*GI and IQ in the control group and a ‘+’ association in the mTBI group).


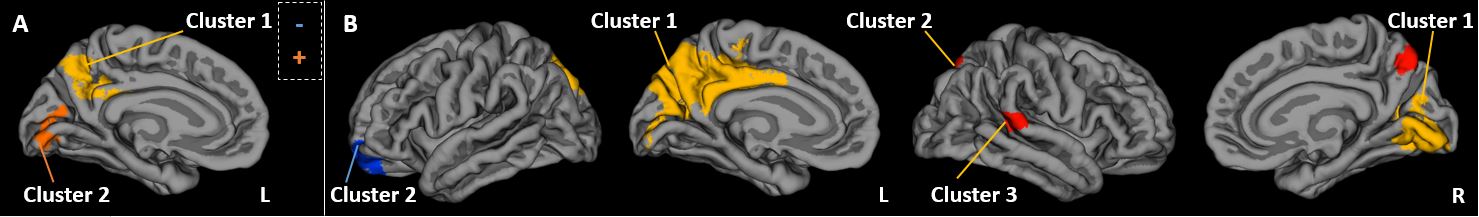


**Figure S2.** (A) Positive *l*GI-IQ association in medial left hemisphere across groups (cluster 1 peak: precuneus, *p*=0.0001; cluster 2 peak: pericalcarine, *p*=0.0013). (B) Group-by-IQ interaction effects: (*L*: cluster 1 peak: precuneus, *p*=0.0001; cluster 2 peak: rostral middle frontal, *p*=0.007; *R*: cluster 1 peak: lingual, *p*=0.0001; cluster 2 peak: precuneus, *p*=0.0263; cluster 3 peak: superior temporal, *p*=0.0291).
