# Supplementary material for: Cortical Gyrification Morphology in Adult Males with Mild Traumatic Brain Injury
Source: Neurotrauma Rep. 2022 Aug 9;3(1):299–307. doi: 10.1089/neur.2021.0032 (PMC9438439; doi:10.1089/neur.2021.0032)
Supplement: Supplemental data [file Supp_FigS1.docx]

**Associations between *l*GI and mTBI severity and symptoms**

Across groups, we found a significant negative association between mTBI severity and *l*GI in a cluster located in the left temporal lobe (cluster 1 peak: fusiform, *p*=0.0001) and a positive association in a cluster located in the left superior parietal lobe (cluster 2 *p*=0.0001, Figure S1, A). The *l*GI-severity correlation does not significantly differ between groups.

Similarly, across groups, we found a significant negative association between mTBI symptoms and *l*GI in a left temporal cluster (peak: parahippocampal) and a positive association in a cluster located in left superior parietal lobe (peak: precuneus, both *p*s=0.0001, Figure S1, B). The *l*GI-symptoms association does not significantly differ between groups.


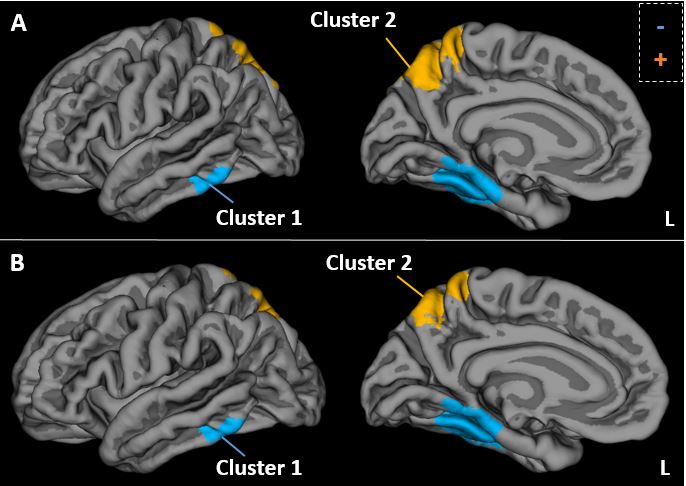


**Figure S1.** (A) *l*GI-severity associations: “-“ association in cluster 1 (peak: fusiform), but a “+” association in cluster 2 (peak: superior parietal, both *p*s=0.0001). (B) *l*GI-symptoms associations: “-“ association in cluster 1 (peak: parahippocampal) but a “+” association in cluster 2 (peak: precuneus, both *p*s=0.0001).
